# Some Natural Photosensitizers and Their Medicinal Properties for Use in Photodynamic Therapy

**DOI:** 10.3390/molecules27041192

**Published:** 2022-02-10

**Authors:** Tomasz Piotr Kubrak, Przemysław Kołodziej, Jan Sawicki, Anna Mazur, Katarzyna Koziorowska, David Aebisher

**Affiliations:** 1 Department of Biochemistry and General Chemistry, The Medical College of The University of Rzeszów, 35-959 Rzeszów, Poland; 2 Department of Biology and Genetics, Medical University of Lublin, Chodzki 4a, 20-093 Lublin, Poland; przemyslawkolodziej@umlub.pl; 3 Department of Analytical Chemistry, Medical University of Lublin, 20-093 Lublin, Poland; jansawicki@umlub.pl; 4 Biochemistry Scientific Club “URCell”, The Medical College of Rzeszów University, 35-959 Rzeszów, Poland; mazuranna09@gmail.com; 5 Biochemistry Science Club “English Division”, The Medical College of Rzeszów University, 35-959 Rzeszów, Poland; kasiakozio@icloud.com; 6 Department of Photomedicine and Physical Chemistry, The Medical College of The University of Rzeszów, 35-959 Rzeszów, Poland; daebisher@ur.edu.pl

**Keywords:** photodynamic therapy (PDT), furanocoumarins, polyacetylenes, thiophenes, tolyporphin, curcumins, alkaloids, anthraquinones

## Abstract

Despite significant advances in early diagnosis and treatment, cancer is one of the leading causes of death. Photodynamic therapy (PDT) is a therapy for the treatment of many diseases, including cancer. This therapy uses a combination of a photosensitizer (PS), light irradiation of appropriate length and molecular oxygen. The photodynamic effect kills cancer cells through apoptosis, necrosis, or autophagy of tumor cells. PDT is a promising approach for eliminating various cancers but is not yet as widely applied in therapy as conventional chemotherapy. Currently, natural compounds with photosensitizing properties are being discovered and identified. A reduced toxicity to healthy tissues and a lower incidence of side effects inspires scientists to seek natural PS for PDT. In this review, several groups of compounds with photoactive properties are presented. The use of natural products has been shown to be a fruitful approach in the discovery of novel pharmaceuticals. This review focused on the anticancer activity of furanocoumarins, polyacetylenes, thiophenes, tolyporphins, curcumins, alkaloid and anthraquinones in relation to the light-absorbing properties. Attention will be paid to their phototoxic and anti-cancer effects on various types of cancer.

## 1. Introduction

Cancer is one of the most frequently occurring diseases and, apart from heart and circulatory system disease, is the greatest threat to health worldwide. It is estimated that, in 2020, almost 20 million cancer cases were reported. Statistically, mortality from malignant neoplasms is about 50%. Today, 30–50% of cancer cases are preventable by reducing risk factors and implementing preventive strategies. The cancer burden can also be reduced through early detection and the appropriate treatment and care of patients who develop cancer. Many cancers have a good chance of being cured if they are diagnosed early and properly treated [1].

The methods of cancer treatment used currently in oncology, such as chemotherapy and radiotherapy, lead to numerous complications related to damage of healthy tissues. Patients often experience local recurrence of the disease or develop secondary tumors via metastases. An inability to selectively act on neoplastic tissue motivates the search for new, more effective methods of treatment of this dangerous disease.

More than 100 years have passed since the discovery and description of photodynamic therapy (PDT). PDT has received widespread interest in recent years as a non-invasive and highly selective approach to cancer treatment.

**Figure 1:**
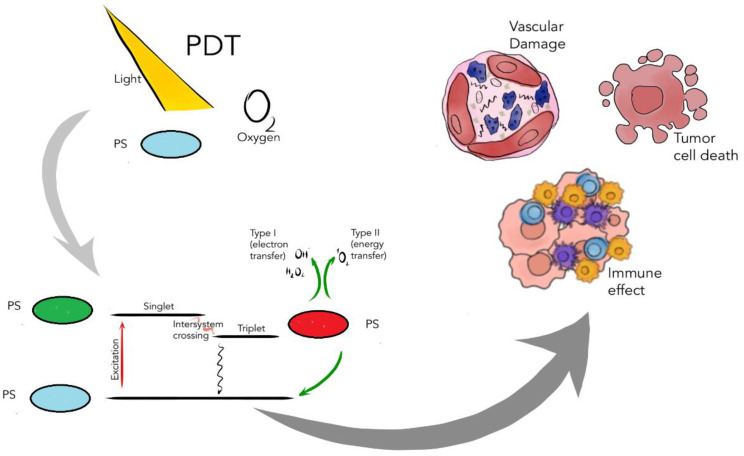
Mechanisms of action of PDT such as inducing immune responses, damaging vascular structures and direct killing of tumor cells. PS’s generate ROS only after exposure to specific light and light activation in the presence of oxygen. Generated ROS mainly cause effector functions (e.g., killing cancer cells, inducing immune responses, damaging vascular structures and direct killing of tumor cells). Following light absorption, the PS reaches an excited singlet state. After an intersystem crossing, the PS, now in a triplet excited state, can react in two ways: react with biomolecules through a hydrogen atom (electron) transfer to form radicals, which react with molecular oxygen to generate ROS (Type I reaction), or the PS in its triplet state can react directly with oxygen through energy transfer, generating singlet oxygen (Type II reaction).

The basis of PDT is to introduce an appropriate photosensitizer (PS) that accumulates in the tumor tissue or local vasculature and then illuminate it with light. The essence of PDT is the destruction of neoplastic cells by reaction with reactive oxygen species such as singlet oxygen or hydroxyl radicals that are generated by photodynamic action. PDT for the treatment of cancer and precancerous conditions it is an alternative treatment but may be used, and usually is, in combination with the other treatment options to traditional methods of treatment (Figure 1).

PDT requires the presence of three basic ingredients. The first component is the presence of PS, i.e., a light-sensitive compound that is delivered to neoplastic tissue. The second is the presence of oxygen (O_2_) dissolved in the tissues, and the third necessary component is the selection of a light source with an appropriate wavelength adapted to the absorption spectrum of a given photosensitizer [2].

The presence of three basic ingredients (PS, O_2_ and light) leads to tumor necrosis [3]. PDT is approved for treatment of neoplastic diseases such as bladder cancer [4,5], skin cancer [6,7], lung cancer [8,9], gastrointestinal tract cancers [10,11,12,13], and cancers of the oral cavity [14,15]. There are also studies on the use of PDT for breast cancer treatment [16,17,18]. To date, PDT has been used successfully in dermatology, gynecology and urology [19].

Historically, Tappeiner and Jesionek were the first to successfully treat skin cancer with eosin in combination with white light. Soon after, Figge demonstrated that hemoporphyrin (HP) has tumor localization properties; Lipson and colleagues initiated PDT in clinical use in the 1960s in the USA [20].

Hematoporphyrin gave rise to the first generation of compounds used as PS in PDT. Currently, second-generation compounds are also used, including synthetic photosensitizers (porphyrin and porphyrin-like compounds). Third-generation drugs combine the high efficiency of the second-generation compounds with greater affinity for tumors, which results in reduced damage to surrounding tissues.

Over the years, many compounds that possess photosensitizing properties have been discovered or synthesized. The search is still ongoing on for photosensitizers that selectively accumulate in neoplastic tissues and do not cause side effects after therapy, e.g., cytotoxicity or mutagenicity. Scientists synthesize new photosensitizers with better biological properties and with absorption in the near infrared (the optimal spectral range for light penetration into tissue) that accumulate in neoplastic tissue and have high quantum yields of reactive oxygen species (ROS). The redox balance is maintained in cancer cells due to their marked antioxidant capacity. 

PDT is used in many fields of medicine, including the treatment of non-oncological diseases and various types of cancer. The use of this therapy in oncology is that the photosensitizer has a tendency to retain in tumor tissues for a longer time compared to normal tissues. The therapy is also used by dermatologists, pulmonologists, urologists and ophthalmologists. It often replaces surgical intervention. Comparing PDT to classical surgery, the recurrence rate may often be higher, but in some respects it is dominant, including requiring no preoperative anesthesia, and healing and tissue regeneration are carried out without scarring and the risk of infection. Moreover, there are not many PDT employs plant-based compounds called photosensitizers (PSs). PSs were discovered from phototoxic plants that were subsequently reported as harmful to human beings and animals over the years [21]. The current literature shows that about 25% of commonly used drugs contain compounds isolated from plants [22].

## 2. Photochemical Reactions in Photodynamic Therapy

**Scheme 1:**
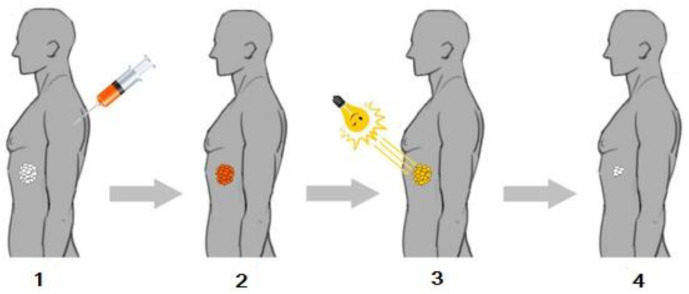
The mechanism of PDT for treatment of cancer. 1. Injection of an appropriate PS; 2. adequate tumor PS concentration; 3. PS laser activation; 4. formation of ROS and tum which occur depending on whether there is a low or high oxygen concentration in the reaction environment of sens or destruction (reproduction from [24]).

A PS is introduced into the patient’s body (intravenously or locally, e.g., on the skin) which accumulates in cancer cells. After a certain period of time, the photosensitizer reaches its maximum concentration in the tumor in relation to the surrounding healthy tissue. After this time, the area is exposed to radiation of wavelength corresponding to the absorption of the PS. The light source must be chosen so that the emission bandwidth of the light source coincides with the PS band absorption necessary for photochemical reaction [23]. The use of PDT in clinical treatment is illustrated in Scheme 1.

As a result of photon absorption, the photosensitizer molecule is excited from the singlet ground state to the excited singlet state. From the point of view of PDT or photodynamic diagnosis (PDD), there are two deactivation pathways that are of importance. The first is transferring excess energy to the environment through the fluorescence process. In PDD, fluorescence detection of a cumulative drug in neoplastic tissue allows for a precise diagnosis with the determination of the shape, size and location of the neoplastic lesion.

The second pathway is intersystem crossing from the exited singlet state to the triplet state. The lifetime of a photosensitizer molecule in the triplet state is sufficiently long to interact with oxygen. Molecular oxygen in the ground state is a triplet and in the process of energy capture it effectively deactivates the triplet state of the PS, in turn generating a strong oxidant known as singlet oxygen [25,26].

**Figure 2:**
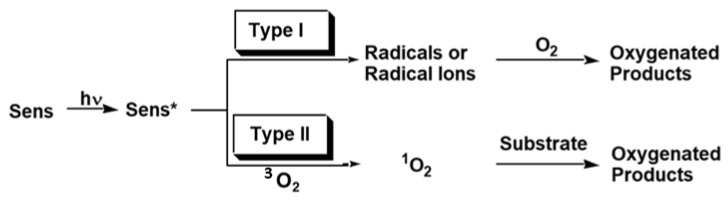
Type I and Type II photosensitized reactions. * Excited state of sens.

The literature describes in detail two mechanisms (Type I and Type II) of the photodynamic effect (Figure 2). 

Type I photosensitized reactions involve electron transfer and lead to the initial formation of radicals and the participation of O_2_ in subsequent steps involved in the oxidation process. Additionally, the Type I mechanism can occur when the environment is hypoxic. The photooxidation reaction leads to the formation of free radicals in tumor tissue. In the type I mechanism, an electron or a hydrogen atom is transferred between the molecule of the excited dye and the neoplastic tissue in which the photochemical reaction takes place. As a result of these reactions, a radical anion or substrate radical is formed.

On the other hand, the type II mechanism involves an initial energy transfer from the triplet excited state of the sensitizer to dissolved O_2_, which is in its ground triplet state.

The Type II mechanism is the dominant mechanism for the destruction of neoplastic tissue. Singlet oxygen (^1^O_2_, refers to the ^1^Δ state) is the predominant type II reactive oxygen species that is able to react with nucleic acids (exclusively guanine), unsaturated lipids and amino acids such as Trp, His and Met. Biological ^1^O_2_ reactions often lead to endoperoxides from [2 + 4] cycloadditions, dioxetanes from [2 + 2] cycloadditions, hydroperoxides from “ene” reactions or phenol oxidations, and sulfoxides from sulfides [27].

Excited singlet oxygen is the result of energy transfer from the triplet form of the photosensitizer molecule to the oxygen molecule. Energy transfer is allowed, and the dye molecule returns to its ground state [28,29,30]. Ideal sensitizer properties and conditions for PDT can lead to cell death in three ways, i.e., apoptosis, necrosis, and autophagy. The response to PDT may differ according to the cell type, its genetic or metabolic potential, and PS intracellular location. The tumor site treated with PDT may initially determine which cell death pathway will be activated. In the beginning, a rescue mechanism is triggered, possibly the process of autophagy. On the other hand, when the PDT damage is sufficiently strong (the concentration of the PS is lower and the incubation time is longer), the cells cannot be repaired and the apoptosis mechanism is activated. If cell damage by PDT is too high (high concentration of photosensitizer and a short incubation time), then it may also lead to necrosis. In this process, proteins involved in both autophagy and apoptosis can be destroyed immediately, and cellular integrity can be broken [31,32].

The EPR (electron paramagnetic resonance) also known as electron magnetic resonance (EMR) or electron spin resonance (ESR), is often used as the “gold standard” in the biological, chemical, and medical systems to detect and characterize radicals. EPR is based on the principle of the absorption of electromagnetic radiation by using electrons with unpaired spins falling under a magnetic field to transition from low to high energy levels, which can be designated as a −1/2 and +1/2, respectively [33].

## 3. The Role of Photosensitizers in PDT

Plant species that are considered toxic are often a source of pharmaceutically active compounds that are some of our drugs and are isolated from plants. Due to the presence of chromophores, they easily absorb light and fluoresce in blue–yellow under long-wave ultraviolet light. These compounds can be produced by the plant as a protective mechanism against a large dose of sunlight, which is why they are often placed in sunscreen and cosmetics for this purpose. Psoralens are typical of the citrus and celery families. Some plants belonging to these groups are known as bladder bushes because the psoralens they contain are known to be phototoxic. This can prove difficult for farmers who remove large amounts of giant hogweed and come into contact with the sap of a psoralen-rich plant, and in the presence of sunlight can cause inflammation and, in severe cases, blistering of the skin. Other species known to be phototoxic are borscht, rue and some *Citruss* spp. A number of culinary-relevant apiaceus herbs, such as celery, parsley, parsnips and angelica, may even be phototoxic due to the presence of furanocoumarins. Psoralens, e.g., form adducts with DNA pyrimidine bases such as thymine via cycloaddition. 

Many plants contain photoactive chemicals and have been identified as phototoxic plants that have the ability to cause skin reactions in humans or animals when they come into contact with the skin followed by exposure to the sun. 

As early as 1942, Klaber [34] observed sunburn in people exposed to sunlight. They then studied many phototoxic plants or phototoxic phytochemicals that, when exposed to sunlight, were susceptible to phototoxic and photogenotoxic skin effects, such as skin irritation, sensitization, allergy, mutations and skin cancers. Photoxins can be found in many different plant families (Table 1), and it follows that the vast majority of phototoxins in the plant kingdom are not related.

Photosensitizers accumulate in cancer cells and, to a lesser extent, in healthy tissues. The effectiveness of PDT largely depends on the properties of the photosensitizer used. A lot of research is carried out to synthesize and optimize the physicochemical properties of photoactive compounds. Often, new photosensitizers of natural origin, e.g., from plants, are obtained. In order for a PS to be used in the diagnosis or treatment of neoplastic diseases, it must meet several conditions [41,42,43,44,45].

1. The PS should selectively accumulate in neoplastic tissue;
2. The PS should preferably be readily available, in the form of a pure compound, and its chemical properties must be carefully established beforehand;
3. The PS should not have phototoxic effects in healthy tissue;
4. The PS should be characterized by a high coefficient of absorption in the spectral range of 600–800 nm, with maximum light penetration through the tissue;
5. The absorption bands of the photosensitizer must not overlap the absorption bands of endogenous dyes such as melanin or hemoglobin and the water absorption bands in the near infrared region;
6. The PS should react efficiently with light to generate singlet oxygen or radicals;
7. The PS and these photoproducts should be characterized by optimal pharmacokinetic properties;
8. The PS should have few side effects;
9. The PS should be of low toxicity and easily excreted from the body to avoid phototoxicity after treatment.

Many dyes, both known and newly synthesized, have been tested for utility in PDT. The requirements that must be met by PS are high and therefore no dye has been discovered that satisfies all of the above criteria [46]. Most PS’s and PS’s photoproducts used in PDT anti-cancer therapy are porphyrin compounds or their derivatives. 

### 3.1. Naturally Occurring Photosensitizers in PDT

Since ancient times, herbal medicine from natural products has been utilized for treating various human ailments. Most current medicines are derived from various medicinal plants, and it is evident that herbal extracts and their compounds should be examined as possible active lead components in cancer drug discoveries. Nature is a valuable reserve for medicinal plants, and many of the pharmaceutically active compounds isolated from medicinal plants have not been tested for photoactive properties. There have been few studies attempting to identify new chemical compounds with photoactivity from plant extracts that can be used as potent natural PSs and in order to achieve the bioavailability and cytotoxicity induction, the drugs are administrated in high concentrations.

Current analytical methods allow for the extraction, purification, determination of the chemical structure, as well as the determination of the pharmacology of active ingredients obtained from natural materials. The natural products are a very useful source for the development of drugs and discovery of new photosensitizers [47,48]. Nature hides a huge amount of pharmaceutically active compounds, some of which are described in the scientific literature.

In recent decades, numerous efforts have been made to isolate new products of natural origin from microbes, plants and other living organisms. Their pharmacological properties are studied, and their anti-cancer properties are assessed. The efforts made have led to the discovery of a number of anti-cancer drugs. It is estimated that in the years 1981–2019, approximately 25% of all newly registered anticancer drugs were derived from natural products [49,50].

In a 2019 review published by Hamblin et al., it is presented that about 400 individual compounds have been recognized as possible candidates for use as PSs [30]. 

In a 2020 review published by Muniyandi et al., more than 300 chemical compounds have already been identified as potential candidates to be used as PSs and the largest group about 700 of anthraquinones possess photoactive properties [48].

In natural extracts, researchers have found new active chemical compounds that display efficient PS properties [51,52,53]. Jong et al. presented the results of screening 2400 extracts from 888 plants for their photosensitizing activity. The study looked at terrestrial plants in the Sarawak forests of Borneo with the goal of finding new photosensitizers for PDT [54]. In 2014, a group of researchers examined the phototoxic properties of 278 extracts. Finally, they described two new PS that showed activity in PDT. Finally, they identified two new photosensitizers with cyclic tetrapyrrole structures [55]. In other studies, the hydrophobic extract of the Arrabidaea chica (Crajiru) plant was presented as a rich collection of PS that could be used in PDT [47]. In 2017, three extracts were tested with the use of MCF-7 breast adenocarcinoma cells for their potential as a source of PS’s in PDT. Photoactivated *L. racemosa* and *A. procera* extracts were found to be more cytotoxic to MCF-7 than the non-tumor human neonatal skin fibroblast lineage [56]. 

**Figure 3:**
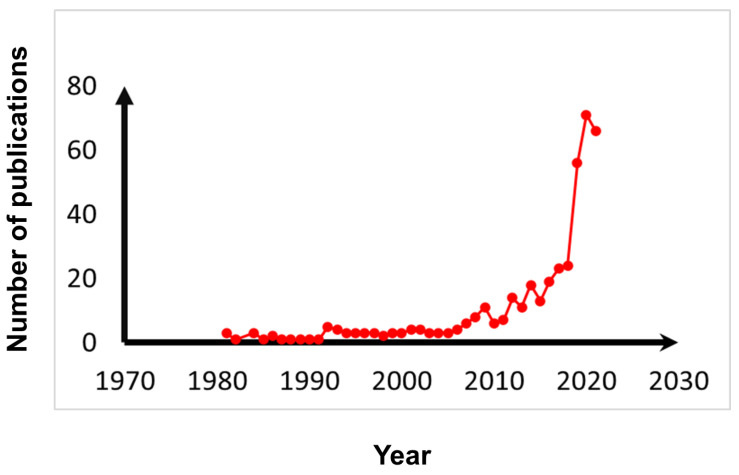
The numbers of publications regarding natural compounds used in PDT.

The above examples and Figure 3 show how important it is to study extracts from various plants for the detection of new PS’s that can be used in PDT. In this brief review, some naturally derived compounds that exhibit PS properties that may be successfully used in PDT are outlined. 

Several PSs were approved for clinical applications or under clinical trials, e.g., Photogem [57], Photodithazine [58], and Photosan [59]. PDT, through the use of PS’s that allow them to interact with many cell receptors and induce cell death, can be used in the treatment of bacterial and viral diseases, autoimmune diseases, and cancer. The antimicrobial PDT has been known for over 30 years and has proven effective against a wide variety of bacteria. Advances in PDT development confirm that it has the ability to kill bacteria, fungi and viruses. ROS generated by the PDT process can deactivate several classes of microbial cells, including Gram-negative bacteria such as Pseudomonas aeruginosa, which are typically characterized by an impermeable outer cell membrane that contains endotoxins and blocks antibiotics, dyes and detergents, protecting the sensitive inner membrane and wall cellular [60]. PDT can be used in infections that do not respond well to antibiotic therapy. There are several features of PDT that make it potentially ideal for treating diabetic feet. The photosensitizer is non-toxic in the dark, but becomes a very effective topical antimicrobial agent when irradiated [61]. PDT was also effective in seventy-seven non-smoking periodontitis patients for acting PDT as an adjuvant treatment. PDT has a positive effect in significantly reducing the periodontal microbial load [62]. The anti-inflammatory and antibacterial action were possible due to the effect of toothpaste containing Polish propolis and plant oils on oral cavity health in patients with oral cleft treated orthodontically [63]. 

**Figure 4:**
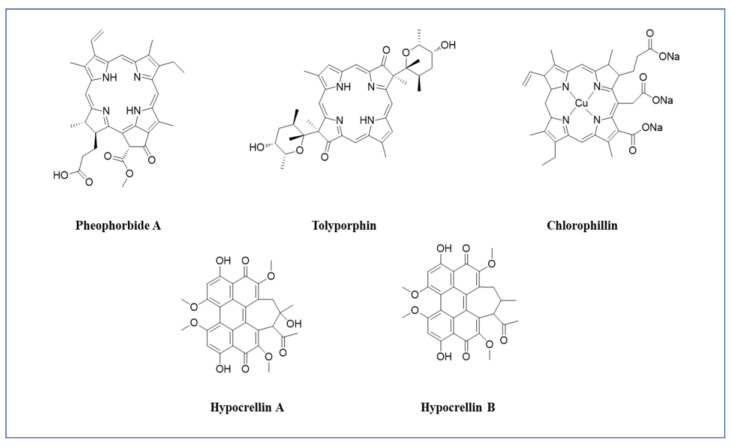
Chemical formulas of the most common photoactive natural PS’s discussed in the review.

**Figure 5:**
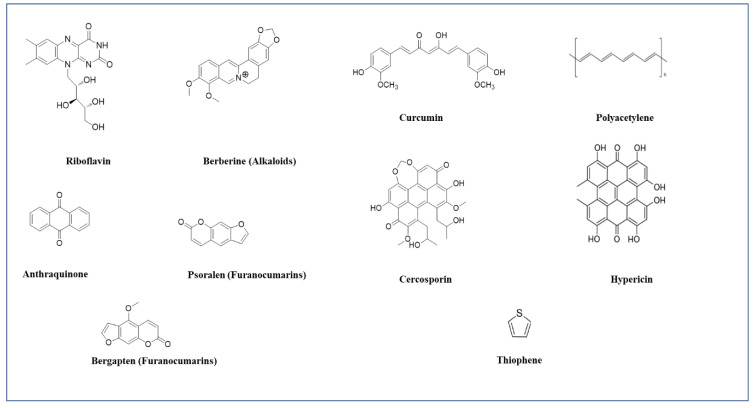
The chemical formulas of the most common photoactive natural PS’s discussed in the review—continued.

The chemical formulas of natural photoactive compounds described in the work are presented in Figure 4 and in Figure 5.

#### 3.1.1. Pheophorbide A


**Absorption Maxima**

**Origin**
670 nmPheophorbide A

For example, *phototrophs* are able to capture the energy in light thanks to photosynthetic pigments, such as chlorophyll and *bacteriochlorophyll**.*** Chlorophyll breakdown products are usually tested for their antioxidant and anti-inflammatory effects. The chlorophyll derivative pheophorbide a (PPBa) is a photosensitizer that can induce significant antiproliferative effects in several human cancer cell lines [64]. This compound is isolated from silkworm excreta [65] and Chinese medicinal herb *Scutellaria barbarta* [66]. Pheophorbide-b methyl ester, 13(2)-hydroxyl (13(2)-S) pheophorbide-a methyl ester and 13(2)-hydroxyl (13(2)-R) pheophorbide-b methyl ester all demonstrated dark cytotoxic activity against leukemia cells with IC(50) values in the range of 46–79 µM, whereas the compound pheophorbide—a methyl ester exhibited only weak dark cytotoxic activity [67].

Many studies report the inhibitory effect of pheophorbide a on the growth of human neoplastic cells. Studies regarding dark toxicities of pheophorbides are performed in hepatocellular carcinoma [68], human uterine cancer [69], glioblastoma multiforme [70] and bladder cancer [71]. PDT studies with the inhibitory effect of pheophorbide are reported in head and neck cancer [72], Barrett’s esophagus [73], leukemia [74], prostate cancer [75,76,77] and esophageal cancer [78].

Overall, the research suggests that PDT in combination with Pheophorbide a with the appropriate wavelength (670 nm) turns out to be a potential therapeutic strategy against many cancers.

#### 3.1.2. Curcumins


**Absorption Maxima**

**Origin**
420–480 nm
*Dicinnamoylmethane, curcumin, curcuminoids, demethoxycurcumin, bisdemethoxycurcumin*


Curcumin has recently been classified as both a PAINS (pan-assay interference compounds) and an IMPS (invalid metabolic panaceas) candidate [79]. 

Curcumin is a photosensitizer that is usually found in ketone and enol forms and is isolated from the Curcuma longa rhizome. Curcuminoids used in PDT significantly inhibited cell viability in breast cancer cell lines [80]. Demethoxycurcumin is known to have the highest anti-proliferative effect [81]; broad pharmacological activity of curcumin has been described, such as antibacterial, antiviral, anti-inflammatory and anti-tumor activities [82].

Koon et al., showed that the biological activity of curcumin can be magnified by the combination of light [83]. Subsequently, Dujiic et al. investigated the photodynamic effect of curcumin as an anticancer drug on a tumor model of A431 (human cell epithelial carcinoma). They showed that tumor growth was significantly inhibited in PDT, whereas the control group showed no reduction in tumor volume [84]. Several studies have been carried out with the use of curcumin in PDT and confirmed its anticancer and antibacterial properties [85,86,87,88]. The antiviral activity of photoactivated curcumin was presented in treatment of norovirus surrogates, feline calicivirus (FCV), and murine norovirus (MNV). Results showed that photoactivated curcumin at 50 µg/mL reduced FCV titers by almost 5 log after incubation at 37 °C for 30 min. Lower antiviral activity (0.73 log TCID_50_/mL reduction) was reported for MNV. At room temperature, curcumin at 5 µg/mL reduced FCV titers by 1.75 log TCID_50_/mL. These results represent a step forward in improving food safety using photoactivated curcumin as an alternative natural additive to reduce viral contamination [89]. 

In addition, it has been shown that various modifications in the chemical structure of curcumin can further improve its properties by enhancing molecular stability or solubility [90,91].

#### 3.1.3. Anthraquinones


**Absorption Maxima**

**Origin**
437 nm
*Polygonum cuspidatum, Heterophyllaea pustulata, H. lycioides Aloe vera, Rheum palmatum, Rumex crispus Polyathia suberosa, Dactylopius coccus, Xanthoria parietina, Drechslera avenae, Ramularia collo-cygni. H. perforatum, Fagopyrum esculentum.*


Anthraquinones are a group of compounds that have been isolated from the leaves and stems of the *Heterophyllaea pustulata* Hook f. (*Rubiaceae*) plant. They show a natural phototoxic effect, and the photosensitizing properties are due to the production of singlet oxygen and/or a superoxide anion radical [92]. Comini et al. [48] anthraquinones such as soranjidiol, soranjidiol 1-methyl ether, rubiadin and rubiadin 1-methyl were found to exhibit PDT activity against human breast cancer cells transfected with caspase-3 (MCF-7) [81]. The AQs were reported as kinase and tyrosinase inhibitors as well as cytotoxicity agents. The M. elliptica AQs such as morindone, soranjidiol and rubiadin were also reported for their antitumor activity against lymphocytic leukemia (P-388) cells. 

Rumie Vittar et al. [93] presented innovative evidence confirming the anti-tumor activity of rubiadin in combination with soranjidiol in in vitro photodynamic therapy, triggering the process of apoptosis in human cancer cells. They showed that the combination can be an effective therapeutic strategy.

#### 3.1.4. Polyacetylene and Thiophenes


**Absorption Maxima**

**Origin**
314–350 nm*Asteraceae* spp., *Heliopsisa, Rudbeckia* spp., *Arnica, Centaurea scabiosa, Tagetes erecta, Porophyllum obscurum, Echinops, Bidens, Ambrosia chamissonis, T. minuta, E. latifolius, E. sgrijissi, Rhaponticum uniflorum.*


Thiophene derivatives are characterized by cytotoxic and phototoxic properties towards neoplastic cells [94]. The cytotoxic properties of the thiophenes were not observed in the absence of light. Polyacetylene and thiophene compounds are activated or excited by light with a wavelength of 314–350 nm [95]. Thiophenes derived from *Echinops latifolius Tausch* root are cytotoxic to human cancer cell lines including A375-S2, HeLa, HL-60, K562 and MCF-7 [96,97,98]. Apart from a strong cytotoxic effect on human tumor cell lines, strong analgesic, anti-inflammatory and antibacterial properties are mentioned. After irradiation with near UV light, α-terthienyl and most of its analogues had significant toxicity, with minimum inhibitory concentrations in the range of 0.02–40 μM. Without irradiation Thiophene compounds had no significant toxicity [99].

#### 3.1.5. Tolyporphin

Cyanobacteria are the source of tolyporphins, a member of the porphyrin family. It locates in the endoplasmic reticulum (ER) and is associated with strong phototoxicity in PDT. After the use of tolyporphins, damage to the nuclear membrane has been observed, which may damage neoplastic cells, and indirectly also affect vascular damage [98,100].

#### 3.1.6. Chlorophyllin

Cyanobacteria are also a source of chlorophyllin, which is a derivative of chlorophyll. Chlorophyllin is mainly localized in lysosomes and mitochondria, whereby cancer cells are destroyed by autophagy and apoptosis in PDT. Studies have shown that chlorophyllin has favorable optical properties (600–670 nm) and solubility in aqueous solutions. It is also readily available and chlorophyllin has been successfully used against cancer cells of the bladder [101,102] and of the MCF7 breast cancer cell line [103].

#### 3.1.7. Hypericin

Hypericin is an anthraquinone derivative, isolated from the herb *Hipericum perforatum*. It is widely used in folk medicine, as an antiviral, antidepressant, natural antibiotic and as an anti-cancer drug [104].

The photodynamic activity of hypericin is attributed to its high cell uptake due to high lipophilicity. It is a molecule that requires a carrier in the form of liposomes, micelles or nanoparticles [105]. Theodossiou et al. investigated the hypericin-PDT mechanism and showed that hypericin accumulates mainly in the membranes of various organelles, especially the mitochondria and the endoplasmic reticulum (ER)–Golgi complex, inducing cell death through both Type I and Type II reactions [106]. In photodynamic therapy with hypericin, the produced reactive oxygen species can cause oxidative damage and destroy neoplastic tumors [107]. According to studies, the main mechanisms of cancer cell death after hypericin-PDT are apoptosis, autophagy and necrosis [108,109].

Due to the low solubility of hypericin and the short absorption wavelength (590 nm), a large number of analogues was synthesized, which improved its physicochemical properties [110]. 

Hypericin-mediated PDT has been used in the treatment of skin [111] cervical [112,113] glioblastoma [114] and bladder tumors [115]. Treatment with activated hypericin of patients with nasopharyngeal carcinoma cells [116] and human pancreatic cells [117] is promising. In summary, hypericin is one of the strongest PS extracted from natural sources [118]. 

Liposomal meso-tetrahydroxyphenyl chlorin derivative (Foslipos^®^) and hypericin was used in 1:1 combination to treat head and neck squamous cell carcinoma (HNSCC) cell lines (UMB-SCC 745 and UMB-SCC). Foslipos^®^-treated cells showed cellular toxicity at the highest concentration (10 µg/mL). In contrast, hypericin was toxic at all concentrations (10–0.6 µg/mL). The combination of photosensitizer was nontoxic at all concentrations (10–0.6 µg/mL). Data have shown that combinatorial use reduces the toxicity of the PS, which may be beneficial in the treatment of PDT [119].

ROS fluorescence was only visible after hypericin and mixture-induced PDT. Cell viability was also more affected with these two treatment options under the selected conditions. Examination of death pathways showed that hypericin-mediated cell death was apoptotic, with mTHPC necrotic and the 1:1 mixture showed features of both [120]. 

#### 3.1.8. Hypocrellin

Hypocrellins are isolated from the parasitic fungi Hypocrella bambusae and *Shiraia bambusicola*, found mainly in Asia. There is hypocrellin a and b which belong to the general class of perylene quinonoid pigments. These compounds have been extensively studied as photosensitizers in PDT. Hypocrellins are characterized by high singlet oxygen quantum efficiency, which has attracted attention as potential PS for PDT. Activated hypocrellins produce reactive oxygen species and hypocrellin radicals, which may contribute to phototoxicity of cells. Hypocrellins have the property of easy chemical modification to increase phototoxicity, pharmacokinetics, solubility and light absorption in the red spectral region [121]. The absorption of the wavelength of the hypocrellins is below 600nm, which is considered a disadvantage as PS. Hypocrellins have an affinity for binding to lipids and may also be located in lysosomal compartments, mitochondria and cell membranes [122]. Cell death occurs through apoptosis or necrosis through peroxidation of lipid membranes [123].

When activated with light, hypocrellins have antiviral and antitumor properties [124], and have been used successfully in cancer phototherapy, including the treatment of skin cancer [125] and the breast cancer [126].

#### 3.1.9. Cercosporin

The toxic effect of cercosporin as PS is the production of singlet oxygen, which causes the formation of free radicals, which in turn damage the cytoplasmic membrane and ultimately cell lysis [127]. Cercospora kikuchii is a source of cercosporin extraction, while related elsinochromes are derived from the *Elsinoe* fungus family [128]. Mastrangelopoulou et al. showed that cercosporin is localized in both the endoplasmic reticulum and mitochondrial membranes and cell death occurs through apoptosis and necrosis [128]. The disadvantage of cercosporin in clinical use is its short activation wavelength (below 532 nm) and poor water solubility. 

The photocytotoxicity of cyclosporine was assessed positively against two human glioblastoma cell lines (T98G and U87) and one breast adenocarcinoma (MCF7) with positive results [129].

Cercosporin is a strong photosensitizer, but with a short activation wavelength, making it mainly suitable for superficial PDT treatments [130].

#### 3.1.10. Riboflavin

Leafy vegetables are a main rich source of riboflavin (vitamin B2), but also mushrooms and dairy products also contain it. It is a promising alternative PS molecule which generates ROS after exposure to blue light. Riboflavin has two peaks in the UVA (360 nm) and blue (440 nm) wavelength regions [131].

Due to its favorable and well-known toxicological and pharmacokinetic properties [105,106], riboflavin is recognized by the FDA as generally safe [132]. Hassan et al. [133,134] reported that riboflavin-PDT may support cisplatin-based chemotherapy by alleviating cisplatin-induced toxicity. Riboflavin increased the sensitivity of cisplatin-resistant tumor cells in a mouse model. Cancer cells entered the path of apoptosis and autophagy under the influence of a riboflavin–PDT adjuvant. The results suggest that in PDT therapy, cisplatin in combination with riboflavin may be a better treatment option in cancer patients than cisplatin alone.

The properties of riboflavin as PS have been used successfully in the treatment of many cancers, i.e., in squamous carcinoma cells (SCC-13) [135], in HeLa cells [136] or in melanoma [137].

#### 3.1.11. Alkaloids

Alkaloids are a diverse group of secondary metabolites obtained from higher plants. The chemical structure classifies them as heterocyclic compounds with a nitrogen atom in the ring. The representative alkaloid considered to be the most photochemical is berberine [138,139]. Numerous studies demonstrate that berberine has anti-cancer properties enhanced by UV radiation and blue light [140,141]. Photoactivation of berberine occurs at a wavelength of 410 nm and has been shown to be effective in controlling the growth of brain cancer cells [142]. Lopes et al. confirmed that berberine has promising potential as a photosensitizer in PDT. Their proposed treatment induced changes in metabolites related to cell proliferation, tumor formation and angiogenesis in renal cell carcinoma (RCC) [142] and in cervical cancer cells (HaCaT) [143].

Thus, they confirmed a significant anti-cancer effect on kidney cancer cells. Activa berberine promotes autophagy and apoptosis by producing ROS [140,141,142,143]. Berberine can be used as a natural PS in PDT applications with minimal side effects [144].

Among the alkaloids, carboline [145] and harmine [146] are distinguished as strong photosensitizers. They are highly photoactive compounds and after irradiation they produce a significant amount of ROS [146].

Jantova et al. confirmed the ability of alkaloids to photosensitize and generate ROS in the presence of a light source [147].

#### 3.1.12. Furanocoumarins

Furanocoumarins are a structurally diverse group of natural substances, i.e., secondary metabolites isolated from higher plants, showing a number of bioactivities, including anti-cancer [148]. 

The most active photoactive substance is psoralen, sensitive to UVA light in the wavelength range of 300–400 nm. It has been shown to be effective in the treatment of psoriasis, dermatitis, eczema and other skin problems [149]. Numerous studies indicate the anti-cancer activity of furanocoumarins against various types of cancer, such as breast, skin and leukemia. When exposed to light, furanocoumarins induce cancer cell death by inhibiting the signal transducer and activator of transcription3 (STAT3), nuclear factor B (NF-B), phosphatidylinositol-3 kinase and AKT protein expression. These pathways play a key role in tumor development by activating several inflammatory genes. Panno et al. investigated the efficacy of furanocoumarins against breast cancer. They found inhibition of tumor growth by inhibiting STAT3 protein expression [150].

On the other hand, the team of Kim et al. [151,152] showed that in leukemia cells, furanocoumarins inactivated JAK (Janus-activated kinase), c-Src and STAT3 proteins and lowered the concentrations of Bcl-xl and Bcl-2 proteins, which are responsible for the apoptosis process. Increased activity of furanocoumarins, bergapten and citroptenhas, also been demonstrated after UV irradiation against malignant melanoma (A375) [153].

Psoralen and its derivatives like 5-methoxypsoralen (5-MOP) and 8-methoxypsoralen (8-MOP) increase cytotoxicity after UV irradiation with a wavelength of 320–400 nm and are effective against cutaneous T-cell lymphoma [154,155].

PUVA (Psoralen Ultra-Violet A) treatment has been shown furocoumarins having a methoxy group to be effective against B16F10 mouse melanoma cells [156].

## 4. Conclusions

Cancer diseases are one of the greatest threats of the modern world. Photodynamic therapy (PDT), due to its advantages, is of interest both as a method of treatment and diagnosis of neoplastic changes, but also as a complementary method, used in parallel with conventional methods. We hope that our review will be helpful to improve the field of anticancer drugs by providing information about biological changes that occur in cells.

An important feature of PDT therapy is that cancer cells cannot become resistant to singlet oxygen. The advantage of photodynamic therapy over chemotherapy, radiotherapy or surgical methods is the selective accumulation of the photosensitizer in the selected tissue and limited activity at the irradiation site. A significant advantage of this method is that no scars are formed, unlike traditional surgical methods. Due to the application of PDT in patients suffering from neoplastic diseases, difficult, multi-stage and very costly surgeries can be avoided.

Substances derived from natural sources are becoming more readily available due to advanced analytical devices. Scientists are discovering new photoactive chemicals from natural extracts that can act as efficient PS.

As the identification of photoactive natural products increases, many new natural PSs are expected to be discovered in the near future. Photodynamic therapy is becoming an increasingly popular therapeutic method that guarantees not only effective treatment, but also diagnosis.

Combinations of PDT with other treatments, such as chemotherapy, radiation therapy, immunotherapy, and even herbal therapy, may be a promising approach for a variety of cancer types for which the results obtained with monotherapy are ineffective.

## Figures and Tables

**Table 1:** Examples of photototoxic plant species and their reported some phototoxins.

Common Name	Plant Species	Phototoxin(s)	References
*Ammi majus* L.	Bishop’s weed	e.g., Xanthotoxin, bergapten, oxypeucedanin	[35]
*Ammi visnaga* (L.)	Apiaceae	e.g., Xanthotoxin; 8-hydroxybergapten; imperatorin	[36]
*Pastinaca sativa*	Wild parsnip	e.g., Xanthotoxin; bergapten; imperatorin	[37]
*Cymopterus watsonii*	*Apiaceae*	e.g., Xanthotoxin; bergapten	[38]
*Cullen cinereum*	Hoary Scurf-pea	e.g., Psoralen	[39]
*Ruta graveolens* L.	Rua	e.g., Psoralen; bergapten; isorutarin	[40]

## Data Availability

Not applicable.
